# Supplementary material for: Multi-Target Alternative Approaches to Promoting Fresh-Cut Carrots’ Bioactive and Fresh-like Quality
Source: Foods. 2022 Aug 12;11(16):2422. doi: 10.3390/foods11162422 (PMC9407139; doi:10.3390/foods11162422)
Supplement: Supplementary file 1 [file foods-11-02422-s001.zip › foods-1830129-supplementary.pdf]

Article

Multi-Target Alternative Approaches to Promoting Fresh-Cut Carrots' Bioactive and Fresh-Like Quality

Carla Alegria[1,2], Elsa M. Gonçalves[3,4*], Margarida Moldão-Martins[5], Marta Abreu[3,5*]

[1] SFCOLAB - Associação Smart Farm COLAB Laboratório Colaborativo para a Inovação Digital na Agricultura, Rua Cândido dos Reis nº1, Espaço SFCOLAB, 2560-312 Torres Vedras, Portugal
[2] cE3c – Centre for Ecology, Evolution and Environmental Changes & CHANGE - Global Change and Sustainability Institute, Faculdade de Ciências, Universidade de Lisboa, 1749-016 Lisboa, Portugal
[3] Unidade Tecnologia e Inovação, Instituto Nacional de Investigação Agrária e Veterinária, I.P.. Av. da República, Quinta do Marquês, 2780-157 Oeiras, Portugal
[4] GeoBioTec—Geobiociências, Geoengenharias e Geotecnologias, Faculdade de Ciências e Tecnologia, Universidade Nova de Lisboa, 2829-516 Caparica, Portugal
[5] LEAF—Linking Landscape, Environment, Agriculture and Food Research Center, Associated Laboratory TERRA, Instituto Superior de Agronomia, Universidade de Lisboa, Tapada da Ajuda, 1349-017 Lisboa, Portugal
* Correspondence: elsa.goncalves@iniav.pt (E.M.G.); marta.abreu@iniav.pt (M.A.)

Supplementary Material

Tables

Supplementary Material Table S1. Factor loadings of the preliminary Principal Component Analysis (PCA) of the full data matrix (56 samples and 13 variables).

Variable	Factor 1	Factor 2	Factor 3	Factor 4
TPC	0.202268	-0.822794	-0.335067	0.077934
CA	0.121653	-0.894850	0.212862	0.168795
PAL	0.061471	-0.859681	0.171582	-0.290313
TCC	0.383605	0.201281	-0.502062	0.177074
AOx	0.179585	-0.552866	-0.633246	0.218847
pH	0.774382	0.142915	-0.380450	0.052821
SSC	0.360808	-0.180590	0.609355	0.501420
Rejection	-0.901081	-0.199603	-0.011145	0.044524
O_2	0.758970	-0.176641	0.133980	-0.392505
CO_2	-0.860636	0.310495	-0.116615	0.320082
TAPC	-0.878943	-0.416320	-0.052911	0.085895
LAB	-0.941584	-0.147247	-0.060289	0.053976
Y&M	-0.688761	-0.031297	-0.140881	-0.569591

Supplementary Material Table S2. Used variable and sample codes for hierarchical cluster analysis and PCA analysis for overall quality assessment.

Variables	Codes
Total phenolic content	TPC
PAL activity	PAL
Chlorogenic Acid	CA
Total aerobic plate counts	TAPC
Lactic acid bacteria counts	LAB
Oxygen concentration	O_2
Carbon dioxide concentration	CO_2
pH	pH
Rejection index (sensorial)	Rejection
**Samples**	**Codes**
Control (untreated) packed in film A_day 0-14	CA0; CA3; CA5; CA7; CA10; CA12; CA14
Control (untreated) packed in film B_day 0-14	CB0; CB3; CB5; CB7; CB10; CB12; CB14
Heat-treated packed in film A_day 0-14	HA0; HA3; HA5; HA7; HA10; HA12; HA14
Heat-treated packed in film B_day 0-14	HB0; HB3; HB5; HB7; HB10; HB12 ;HB14
UV-treated packed in film A_day 0-14	UA0; UA3; UA5; UA7; UA10; UA12; UA14
UV-treated packed in film B_day 0-14	UB0; UB3; UB5; UB7; UB10; UB12; UB14
HS x UV-treated packed in film A_day 0-14	HUA0; HUA3; HUA5; HUA7; HUA10; HUA12; HUA14
HS x UV-treated packed in film B_day 0-14	HUB0; HUB3; HUB5; HUB7; HUB10; HUB12; HUB14

Supplementary Material Table S3. Changes in atmosphere composition (O_2 and CO_2), total phenolic content (TPC), chlorogenic acid content (CA), PAL activity (PAL), pH, total aerobic plate count (TAPC), lactic acid bacteria (LAB) and yeast and moulds (Y&M) and rejection index (sensorial) of shredded carrot samples as affected by abiotic stress treatments and MAP conditions during low-temperature storage (5 °C, 14 days).

Treatment	Packaging film	Storage (days)	O_2	CO_2	TPC	CA	PAL	pH	TAPC	LAB	Y&M	Rejection
C	A	0	21.0^{r}±0.0	0.0^{a}±0.0	57.67^{cdefghi}±3.23	2.90^{abcde}±0.01	30.47 ^{abcd}±2.05	6.2^{opqr}±0.0	4.3^{defgh}±0.2	2.6^{cdefgh}±0.1	3.2^{bcdefghi}±0.2	1.2^{ab}±0.4
		3	0.5^{ab}±0.5	20.3^{klmno}±0.9	61.10^{efghi}±3.32	2.64^{ab}±0.02	72.57 ^{defgh}±8.94	5.8^{klmnop}±0.1	6.1^{ijkl}±0.1	4.9^{ijklmn}±0.1	3.9^{defghij}±0.2	1.2^{ab}±0.3
		5	0.0^{a}±0.1	28.8^{rs}±1.2	81.60^{ijklm}±2.25	3.04^{abcdefg}±0.07	24.50^{abc}±1.65	5.5^{ghijk}±0.1	6.9^{jklm}±0.1	6.0^{mno}±0.1	3.7^{cdefghij}±0.3	2.4^{de}±0.3
		7	0.1^{a}±0.2	30.0^{s}±1.7	86.97^{jklm}±14.67	3.04^{abcdefg}±0.16	25.07^{abc}±1.55	6.3^{r}±0.0	7.6^{lmno}±0.1	7.0^{opqu}±0.0	3.4^{cdefghij}±0.4	3.6^{hij}±0.4
		10	0.0^{a}±0.0	38.1^{t}±0.9	42.63^{abcde}±4.63	2.50^{ab}±0.04	8.77^{a}±1.06	5.3^{efghi}±0.1	8.2^{mno}±0.2	8.2^{quv}±0.2	4.0^{efghij}±0.4	3.9^{jk}±0.4
		12	0.0^{a}±0.0	39.3^{t}±1.4	35.83^{abcd}±0.21	3.01^{abcdefg}±0.04	12.40^{a}±1.61	4.7^{bc}±0.0	8.6^{no}±0.1	8.5^{uv}±0.1	4.4^{ghij}±0.3	4.4^{kl}±0.3
		14	0.0^{a}±0.1	42.3^{t}±0.9	32.80^{ab}±0.66	2.31^{a}±0.06	11.83^{a}±1.42	4.2^{a}±0.0	8.9^{o}±0.1	8.8^{v}±0.1	4.8^{ij}±0.1	4.9^{l}±0.2
	B	0	21.0^{r}±0.0	0.0^{a}±0.0	56.33^{bcdefgh}±2.20	2.90^{abcdef}±0.03	31.30^{abcde}±2.95	6.2^{pqr}±0.1	4.1^{cdefg}±0.1	2.6^{cdefgh}±0.2	3.3^{bcdefghi}±0.1	1.1^{a}±0.2
		3	7.5^{efghijkl}±1.8	10.3^{efgh}±0.5	67.97^{fghijk}±6.38	4.31^{hij}±0.02	212.57^{pq}±26.76	5.8^{jklmno}±0.0	6.1^{ijkl}±0.3	4.9^{ijklm}±0.3	4.3^{fghij}±0.0	1.2^{ab}±0.3
		5	6.3^{cdefghij}±1.6	13.2^{ghi}±1.4	89.63^{klmn}±7.22	3.62^{cdefghi}±0.02	129.93^{jklm}±17.57	5.5^{fghijk}±0.0	7.1^{klmn}±0.2	5.6^{klmno}±0.3	4.1^{efghij}±0.2	2.2^{k}±0.3
		7	4.0^{abcdefg}±0.9	16.5^{ijk}±2.2	111.93^{nopq}±20.46	6.00^{mn}±0.09	165.03^{mno}±8.89	6.1^{mnopqr}±0.0	8^{mno}±0.2	6.3^{mnop}±0.2	3.9^{defghij}±0.4	3.1^{fghi}±0.2
		10	2.1^{abcd}±0.8	19.1^{jklm}±1.4	98.63^{lmno}±12.88	7.61^{p}±0.55	232.00^{pqr}±8.18	5.6^{hijkl}±0.1	8.9^{o}±0.3	6.8^{opqu}±0.4	5.2^{j}±0.2	3.5^{hij}±0.4
		12	1.5^{abc}±0.6	18.9^{jklm}±1.6	80.20^{hijklm}±15.80	7.46^{op}±0.18	76.23^{efghi}±9.86	5.4^{efghij}±0.0	8.9^{o}±0.2	6.7^{nopq}±0.3	5.2^{j}±0.2	4.2^{k}±0.3
		14	5.2^{bcdefghi}±4.3	14.8^{hij}±4.8	102.67^{mnop}±12.80	7.97^{p}±0.19	268.87^{r}±14.14	4.8^{cd}±0.2	8.9^{o}±0.1	6.6^{nopq}±0.2	5.2^{j}±0.3	4.9^{l}±0.2
H	A	0	21.0^{r}±0.0	0.0^{a}±0.0	53.87^{bcdef}±2.65	2.70^{abc}±0.05	27.13^{abc}±2.20	6.3^{qr}±0.1	2.6^{abc}±0.1	<10^{1} CFU.g^{-1}	<10^{1} CFU.g^{-1}	1.0^{a}±0.0
		3	2.2^{abcd}±0.2	6.3^{bcde}±1.7	68.10^{fghijk}±8.16	2.73^{abc}±0.01	63.43^{cdefg}±7.99	6.0^{lmnopqr}±0.0	3.7^{bcdef}±0.5	1.4^{abc}±0.1	<10^{1} CFU.g^{-1}	1.1^{a}±0.2
		5	1.1^{ab}±0.1	8.7^{cdefg}±0.9	88.40^{klmn}±8.91	2.80^{abcd}±0.03	18.80^{abc}±2.33	6.1^{mnopqr}±0.1	4.6^{defgh}±0.5	2.2^{bcdefg}±0.6	<10^{1} CFU.g^{-1}	1.3^{ab}±0.2
		7	2.6^{abcde}±1.6	16.8^{ijkl}±0.8	97.17^{lmno}±1.79	2.93^{abcdefg}±0.09	20.60^{abc}±1.47	6.4^{r}±0.0	5.5^{ghij}±0.2	2.9^{cdefgh}±0.4	<10^{1} CFU.g^{-1}	1.5^{abc}±0.4
		10	1.8^{abcd}±1.1	23.3^{mnopq}±1.7	55.90^{bcdefg}±2.09	3.32^{bcdefg}±0.10	35.77^{abcde}±4.68	6.3^{qr}±0.1	6.2^{ijkl}±0.6	3.5^{defghi}±1.7	<10^{1} CFU.g^{-1}	2^{cd}±0.3
		12	0.1^{a}±0.1	23.6^{mnopqr}±0.5	34.67^{abcd}±1.50	3.32^{bcdefg}±0.02	18.70^{abc}±0.30	5.4^{efghij}±0.0	6.3^{ijkl}±0.5	4.4^{hijkl}±0.6	<10^{1} CFU.g^{-1}	2.5^{de}±0.2
		14	0.1^{a}±0.0	25.3^{opqrs}±2.0	33.07^{ab}±1.01	2.89^{abcde}±0.05	26.47^{abc}±1.95	5.4^{efghij}±0.2	6.4^{ijkl}±1.3	5.3^{jklmno}±0.7	<10^{1} CFU.g^{-1}	2.9^{efg}±0.2
	B	0	21.0^{r}±0.0	0.0^{a}±0.0	55.40^{bcdefg}±3.51	3.70^{defghi}±0.03	34.37^{abcde}±2.11	6.2^{pqr}±0.1	2.4^{ab}±0.3	0.7^{ab}±0.6	<10^{1} CFU.g^{-1}	1.0^{a}±0.0
		3	14.3^{opq}±0.4	6.1^{bcde}±0.1	50.77^{abcdef}±6.43	3.77^{efghi}±0.01	159.87^{lmn}±29.54	6.0^{lmnopqr}±0.1	3.5^{bcd}±0.6	1.9^{bcde}±0.3	<10^{1} CFU.g^{-1}	1.1^{a}±0.2
		5	9.5^{hijklmno}±1.9	9.0^{cdefg}±0.7	94.30^{lmno}±3.60	5.32^{klm}±0.10	60.37^{bcdefg}±3.84	6.0^{lmnopqr}±0.2	4.5^{defgh}±0.5	3.1^{defghi}±0.6	<10^{1} CFU.g^{-1}	1.2^{ab}±0.2
		7	12.7^{nopq}±2.3	7.1^{bcde}±1.7	133.67^{qrst}±6.65	9.14^{q}±0.21	115.27^{hijkl}±16.88	6.2^{pqr}±0.1	5.5^{ghij}±1.0	4.2^{hijkl}±1.6	<10^{1} CFU.g^{-1}	1.3^{ab}±0.4
		10	11.0^{jklmnop}±0.6	8.1^{bcdefg}±0.1	143.17st±4.46	11.77^{r}±0.17	144.67^{klm}±24.82	6.0^{lmnopqr}±0.2	6.9^{jklm}±0.3	3.6^{efghij}±0.1	<10^{1} CFU.g^{-1}	1.7^{bc}±0.3
		12	11.7^{klmnopq}±0.3	8.9^{cdefg}±0.5	94.40^{lmno}±0.26	15.97^{t}±1.06	217.50^{pq}±22.12	6.1^{nopqr}±0.1	7.1^{klmn}±0.5	3.5^{defghi}±0.6	<10^{1} CFU.g^{-1}	2.1^{cd}±0.2
		14	13.6^{nopq}±3.7	6.5^{bcde}±2.8	117.63^{opqr}±0.47	21.16^{u}±0.98	201.90^{nop}±4.95	5.2^{defg}±0.0	7.2^{klmn}±0.6	3.4^{defghi}±0.6	<10^{1} CFU.g^{-1}	2.5^{de}±0.4
U	A	0	21.0^{r}±0.0	0.0^{a}±0.0	53.97^{bcdef}±2.06	2.63^{ab}±0.02	53.57^{abcdef}±9.28	6.2^{opqr}±0.0	3.9^{bcdef}±0.2	2.3^{bcdefg}±0.2	3.2^{bcdefghi}±0.1	1.0^{a}±0.0
		3	1.3^{abc}±2.1	22.0^{lmnop}±2.7	58.17^{defghi}±10.34	2.63^{ab}±0.05	74.73^{defgh}±8.66	5.9^{klmnopq}±0.1	5.8^{hijk}±0.1	5^{ijklmn}±0.2	4.3^{fghij}±0.2	1.2^{ab}±0.3
		5	0.4^{ab}±0.6	27.8^{qrs}±1.4	78.93^{ghijklm}±8.51	2.99^{abcdefg}±0.03	27.20^{abc}±4.46	6.0^{lmnopqr}±0.1	6.5^{ijkl}±0.1	5.9^{mno}±0.3	3.8^{defghij}±0.2	2.2^{d}±0.2
		7	0.4^{ab}±0.2	30.2^{s}±3.7	81.77^{ijklm}±9.28	3.15^{abcdefg}±0.03	25.23^{abc}±1.31	6.3^{r}±0.1	7.1^{klmn}±0.2	6.8^{opqu}±0.3	3.2^{bcdefghi}±0.2	3.2^{fghi}±0.3
		10	0.0^{a}±0.0	38.6^{t}±1.5	44.17^{abcdef}±1.68	3.03^{abcdefg}±0.02	26.23^{abc}±1.70	5.7^{jklmn}±0.2	8.1^{mno}±0.2	8.0^{pquv}±0.5	4.0^{efghij}±0.0	3.6^{ij}±0.2
		12	0.0^{a}±0.0	38.4^{t}±1.4	40.10^{abcde}±0.36	3.20^{abcdefg}±0.04	25.00^{abc}±2.86	5.4^{efghij}±0.0	8.4^{no}±0.1	8.3^{uv}±0.3	4.3^{fghij}±0.2	4.0^{k}±0.2
		14	0.0^{a}±0.0	43.1^{t}±1.2	53.03^{abcdef}±0.06	3.13^{abcdefg}±0.12	26.63^{abc}±4.85	4.3^{ab}±0.0	8.6^{no}±0.1	8.5^{uv}±0.2	4.5^{ghij}±0.3	4.4^{kl}±0.4
	B	0	21.0^{r}±0.0	0.0^{a}±0.0	56.17^{bcdefgh}±2.53	3.84^{ghi}±0.14	62.87^{cdefg}±8.14	6.2^{opqr}±0.0	3.6^{bcde}±0.2	1.9^{bcdef}±0.1	3.0^{bcdefgh}±0.2	1.0^{a}±0.0
		3	10.5^{jklmnop}±2.3	8.0^{bcdefg}±1.4	56.70^{bcdefgh}±1.32	4.49^{ijk}±0.27	214.13^{pq}±25.03	5.9^{klmnopq}±0.1	5.8^{hijk}±0.3	3.8^{ghij}±0.1	3.6^{cdefghij}±0.5	1.2^{ab}±0.3
		5	12.9^{nopq}±2.3	7.4^{bcdef}±2.0	78.20^{ghijkl}±1.21	5.70^{lmn}±0.09	103.03^{ghijk}±14.38	5.8^{jklmno}±0.1	6.5^{ijkl}±0.3	3.9^{ghijk}±0.5	2.8^{bcdefgh}±0.5	2.2^{d}±0.2
		7	1.7^{abc}±0.6	18.4^{jklm}±0.8	88.50^{klmn}±5.00	8.17^{p}±0.28	133.70^{jklm}±19.91	6.1^{mnopqr}±0.0	7.2^{klmn}±0.5	4.0^{ghijk}±1.2	1.9^{bc}±1.5	3.1^{fghi}±0.2
		10	15.2^{pq}±1.5	5.1^{abcd}±0.6	135.27^{qrst}±13.41	10.00^{q}±0.22	250.73^{qr}±16.38	5.7^{ijklm}±0.2	8.6^{no}±0.2	5.8^{lmno}±0.3	4.6^{hij}±0.1	3.3^{ghi}±0.3
		12	9.8^{ijklmno}±4.3	9.3^{cdefg}±4.0	91.53^{klmn}±8.80	11.43^{r}±0.06	217.13^{pq}±23.91	5.1^{cde}±0.6	8.7^{o}±0.3	5.8^{lmno}±0.5	4.8^{ij}±0.5	3.8^{jk}±0.2
		14	1.4^{abc}±0.5	18.8^{jklm}±1.0	99.47^{lmno}±15.94	9.29^{q}±0.28	220.20^{pq}±21.67	5.0^{cde}±0.1	8.8^{o}±0.3	5.7^{klmno}±0.8	4.9^{ij}±0.3	4.2^{k}±0.3
HU	A	0	21.0^{r}±0.0	0.0^{a}±0.0	52.67^{abcdef}±1.62	4.42^{ijk}±0.10	39.83^{abcde}±7.48	6.2^{pqr}±0.1	1.9^{a}±0.4	<10^{1} CFU.g^{-1}	<10^{1} CFU.g^{-1}	1.0^{a}±0.0
		3	3.4^{abcdef}±0.4	12.4^{fghi}±0.4	63.60^{efghij}±1.97	3.41^{bcdefgh}±0.03	92.33^{fghij}±7.34	6.0^{lmnopqr}±0.0	4.1^{cdefg}±0.3	1.5^{abc}±0.4	<10^{1} CFU.g^{-1}	1.1^{a}±0.2
		5	2.4^{abcd}±1.5	19.7^{jklmn}±2.5	78.37^{ghijkl}±3.46	3.82^{fghi}±0.05	31.33^{abcde}±3.02	6.2^{opqr}±0.1	4.7^{defgh}±0.6	1.9^{bcde}±0.2	<10^{1} CFU.g^{-1}	1.3^{ab}±0.2
		7	4.6^{abcdefgh}±0.1	18.8^{jklm}±0.7	123.77^{pqrs}±5.31	4.44^{ijk}±0.18	16.03^{ab}±2.14	6.3^{r}±0.0	5.2^{fghi}±1.2	2.3^{bcdefg}±0.3	<10^{1} CFU.g^{-1}	1.5^{abc}±0.4
		10	0.5^{ab}±0.5	25.2^{opqrs}±1.2	48.77^{abcdef}±4.11	6.04^{mn}±0.07	35.17^{abcde}±4.20	6.2^{pqr}±0.1	5.7^{hijk}±0.3	3.7^{fghij}±0.3	<10^{1} CFU.g^{-1}	2^{cd}±0.3
		12	0.3^{ab}±0.3	27.2^{pqrs}±2.7	28.97^{a}±1.60	3.39^{bcdefgh}±0.05	26.23^{abc}±3.69	5.5^{ghijk}±0.3	6.4^{ijkl}±0.6	5.1^{ijklmn}±0.2	<10^{1} CFU.g^{-1}	2.6^{ef}±0.2
		14	1.4^{abc}±0.5	24.7^{nopqr}±0.4	33.80^{abc}±4.10	3.01^{abcdefg}±0.07	23.00^{abc}±3.03	5.1^{cdef}±0.0	7.0^{jklm}±1.0	6.4^{mnop}±0.3	<10^{1} CFU.g^{-1}	3.1^{fgh}±0.3
	B	0	21.0^{r}±0.0	0.0^{a}±0.0	50.40^{abcdef}±0.61	5.06^{jkl}±0.04	50.00^{abcdef}±5.71	6.2^{opqr}±0.0	1.7^{a}±0.4	<10^{1} CFU.g^{-1}	<10^{1} CFU.g^{-1}	1.0^{a}±0.0
		3	15.1^{pq}±2.3	5.2^{abcde}±1.8	62.47^{efghi}±5.31	5.18^{jklm}±0.05	206.97^{opq}±38.70	6.0^{lmnopqr}±0.1	3.5^{bcde}±0.5	1.9^{bcd}±0.2	<10^{1} CFU.g^{-1}	1.1^{a}±0.2
		5	13.5^{nopq}±4.0	6.1^{bcde}±2.6	90.20^{klmn}±2.27	6.62^{no}±0.07	120.43^{ijklm}±16.85	6.0^{lmnopqr}±0.0	4.3^{defgh}±0.5	2.6^{cdefgh}±0.4	<10^{1} CFU.g^{-1}	1.2^{ab}±0.2
		7	16.5^{qr}±0.7	3.1^{ab}±0.7	134.00^{qrst}±5.92	8.13^{p}±0.31	145.63^{klm}±26.36	6.2^{opqr}±0.1	5.1^{efghi}±0.5	3.3^{defghi}±0.6	<10^{1} CFU.g^{-1}	1.3^{ab}±0.4
		10	8.9^{ghijklmn}±2.1	9.8^{defgh}±1.0	153.10^{t}±6.08	13.43^{s}±0.17	217.20^{pq}±8.40	6.0^{lmnopqr}±0.1	6.4^{ijkl}±0.8	4.3^{hijkl}±0.9	<10^{1} CFU.g^{-1}	1.7^{bc}±0.3
		12	13.2^{nopq}±0.2	7.7^{bcdef}±0.3	141.67^{rst}±1.65	15.67^{t}±0.72	197.60^{nop}±10.10	5.7^{jklmn}±0.1	6.9^{jklm}±0.5	5.1^{ijklmn}±0.4	<10^{1} CFU.g^{-1}	2.2^{d}±0.2
		14	12.5^{mnopq}±0.3	7.0^{bcde}±0.9	94.30^{lmno}±12.75	13.80^{s}±0.66	219.07^{pq}±10.97	5.3^{efgh}±0.1	7.3^{klmn}±0.3	5.8^{lmno}±0.3	<10^{1} CFU.g^{-1}	2.7^{ef}±0.4

Treatment: **C**- Control (untreated); **H**- Heat shock (100 °C/45 s); **U**- UV-C (2.5 kJ.m^{-2}); **HU**- Heat shock (100 °C/45 s) x UV-C (2.5 kJ.m^{-2}); **Packaging film**: **A**-no microperforations; **B**- laser established micro-perforations at 120 µm intervals (PPlus® - 35PA120).
Variables units: O_2- %; **CO_2**- %; **TPC** –mg CAE.100 g^{-1}; **CA** – mg.100 g^{-1}; **PAL**- µmol t-cinnamic acid.h^{-1}.100 g^{-1}; **TAPC, LAB, Y&M** – Log_{10} CFU.g^{-1}; **Rejection** –hedonic scale.
Values are mean of five replicates±SD. In the same column, different letters represent significant differences at p=0.05 (Tukey HSD test).

Figures

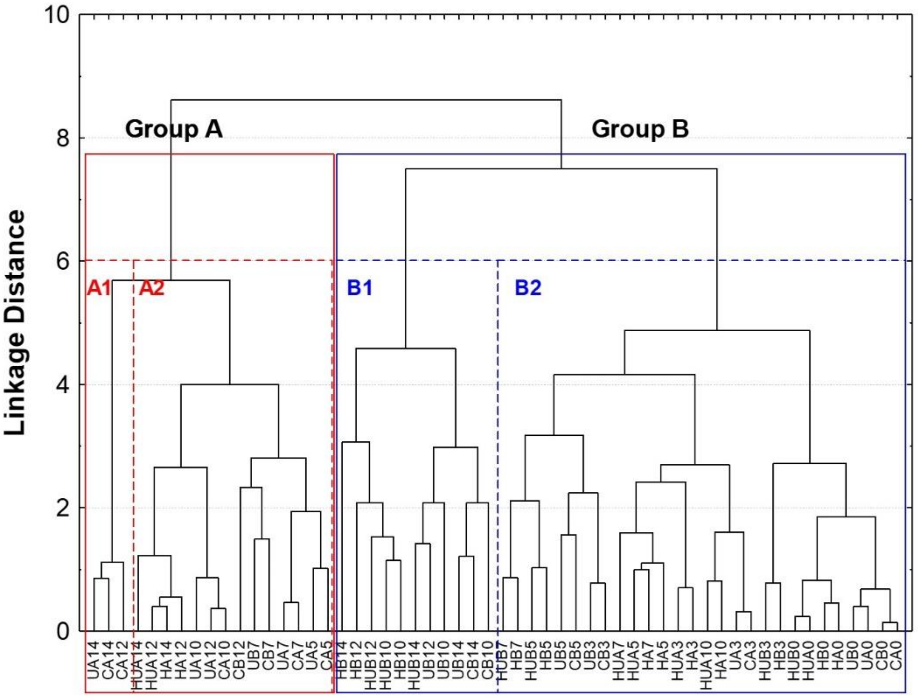

Supplementary Material Figure S1. Hierarchical cluster analysis dendrogram of the data matrix.
